# Chitotetraose activates the fungal-dependent endosymbiotic signaling pathway in actinorhizal plant species

**DOI:** 10.1371/journal.pone.0223149

**Published:** 2019-10-10

**Authors:** Mireille Chabaud, Joëlle Fournier, Lukas Brichet, Iltaf Abdou-Pavy, Leandro Imanishi, Laurent Brottier, Elodie Pirolles, Valérie Hocher, Claudine Franche, Didier Bogusz, Luis G. Wall, Sergio Svistoonoff, Hassen Gherbi, David G. Barker

**Affiliations:** 1 Laboratory of Plant-Microbe Interactions (INRA/CNRS/University of Toulouse), Castanet-Tolosan, France; 2 Laboratory of Biochemistry, Microbiology and Soil Biological Interactions, Department of Science and Technology, National University of Quilmes, CONICET, Bernal, Argentina; 3 Laboratory of Tropical and Mediterranean Symbioses (IRD/INRA/CIRAD/University of Montpellier/Supagro), Montpellier, France; 4 Plant Diversity, Adaptation and Development (IRD/University of Montpellier), Montpellier, France; University of Nebraska-Lincoln, UNITED STATES

## Abstract

Mutualistic plant-microbe associations are widespread in natural ecosystems and have made major contributions throughout the evolutionary history of terrestrial plants. Amongst the most remarkable of these are the so-called root endosymbioses, resulting from the intracellular colonization of host tissues by either arbuscular mycorrhizal (AM) fungi or nitrogen-fixing bacteria that both provide key nutrients to the host in exchange for energy-rich photosynthates. Actinorhizal host plants, members of the Eurosid 1 clade, are able to associate with both AM fungi and nitrogen-fixing actinomycetes known as *Frankia*. Currently, little is known about the molecular signaling that allows these plants to recognize their fungal and bacterial partners. In this article, we describe the use of an *in vivo* Ca^2+^ reporter to identify symbiotic signaling responses to AM fungi in roots of both *Casuarina glauca* and *Discaria trinervis*, actinorhizal species with contrasting modes of *Frankia* colonization. This approach has revealed that, for both actinorhizal hosts, the short-chain chitin oligomer chitotetraose is able to mimic AM fungal exudates in activating the conserved symbiosis signaling pathway (CSSP) in epidermal root cells targeted by AM fungi. These results mirror findings in other AM host plants including legumes and the monocot rice. In addition, we show that chitotetraose is a more efficient elicitor of CSSP activation compared to AM fungal lipo-chitooligosaccharides. These findings reinforce the likely role of short-chain chitin oligomers during the initial stages of the AM association, and are discussed in relation to both our current knowledge about molecular signaling during *Frankia* recognition as well as the different microsymbiont root colonization mechanisms employed by actinorhizal hosts.

## Introduction

A limited number of soil microorganisms have acquired the remarkable capacity to colonize plant roots and form mutually beneficial endosymbiotic associations. The most widespread of these are the obligate biotrophic fungi belonging to the Glomeromycota phylum, able to associate with the majority of terrestrial plants resulting in the formation of highly ramified intracellular root cortical structures known as arbuscules [1,2]. Arbuscular mycorrhizal (AM) fungi scavenge vital soil nutrients such as phosphorus, which are then delivered to the host plant *via* the elaborate symbiotic interface created within arbuscule-containing cortical cells, in exchange for energy-rich metabolites. The AM association is thought to have contributed to the successful establishment of the earliest terrestrial plant species over 400 million years ago [3].

In addition to this ubiquitous fungal symbiosis, certain dicot species belonging to the Eurosid 1 clade subsequently evolved additional beneficial associations with nitrogen-fixing soil bacteria. Gram-negative rhizobia primarily associate with legume Fabales hosts, whilst gram-positive filamentous actinobacteria known as *Frankia* are able to colonize actinorhizal host species belonging to the Fagales, Cucurbitales and Rosales orders. In the majority of cases the nitrogen-fixing bacteria multiply and differentiate intracellularly within *de novo* specialized plant organs known as root nodules, thereby providing a valuable source of nitrogen to the host in return for photosynthate-derived resources. Interestingly, initial root colonization by nitrogen-fixing bacteria can be either intra- or intercellular depending upon the host plant [4]. Whilst intracellular root entry *via* infection thread compartments constructed within root hairs has been well-documented in model legume hosts such as *Medicago truncatula* [5,6], intercellular modes of colonization in legumes are only poorly understood at the molecular/cellular levels. Both types of root colonization by *Frankia* have also been observed for actinorhizal hosts [4,7]. For example, intracellular entry *via* root hairs appears to be common amongst Fagales species such as *Alnus glutinosa* or *Casuarina glauca*, whereas intercellular modes of root colonization appear to be predominant for Rosales species typified by *Ceanothus* spp, *Elaeagnus angustifolia* or *Discaria trinervis* [8–10]. In the case of initial root colonization by AM fungi, experiments performed on both legume and non-legume hosts have demonstrated that AM fungal hyphae traverse atrichoblast (non-root hair) cells of the root epidermis within host-constructed transcellular compartments by a mechanism reminiscent of root hair infection thread-mediated colonization by nitrogen-fixing bacterial symbionts [2,3].

An important parameter for the establishment of both AM and nitrogen-fixing endosymbioses is the capacity for mutual recognition of the respective partners. In particular, it is now well-established that host plant perception of appropriate secreted microbial molecules leads to the activation of a Conserved Symbiotic Signaling Pathway (CSSP) required for subsequent microbe root entry [11,12]. A characteristic feature of the CSSP is the triggering of sustained nuclear-associated calcium oscillations which can be conveniently monitored *in vivo* using fluorescent Ca^2+^ reporters [13,14]. For the large majority of rhizobial-legume associations, decorated lipo-chitooligosaccharides (LCOs) known as nodulation (Nod) factors play this key signaling function, and these LCOs are specifically recognized by host Lysin Motif (LysM) receptor like kinase (RLK) receptor complexes [15,16]. Based on research on legumes such as *M*. *truncatula*, there is evidence that chitin-based molecules are also involved in pre-infection AM fungal–host signaling. These include both AM fungal LCOs known as Myc LCOs [17] and AM fungal short-chain chitin oligomers (referred to collectively as Myc COs) and typified by chitotetraose (CO4) [18]. Although the precise roles of these chitinaceous factors remain to be determined, recent studies focused on the monocot rice have confirmed the importance of CO4 in AM fungal-host signaling [19], and identified the LysM RLK OsCERK1 as a putative component of the host perception machinery for these fungal-secreted molecules [20].

In contrast with *Rhizobium*-legume associations, relatively little is known about pre-infection microsymbiont-host signaling during the establishment of actinorhizal symbioses. Although strong evidence suggests that intracellular-colonizing *Frankia* which nodulate *A*. *glutinosa* and *C*. *glauca* do secrete symbiotic signaling factors, the precise chemical nature of these molecules has not yet been determined. The use of root hair deformation assays, nuclear-localized Ca^2+^ reporters to monitor CSSP activation and the expression of symbiotic reporter genes have together led to the conclusion that these *Frankia* make use of alternative non-chitinaceous signals [21–23]. On the other hand, it is currently unknown whether chitin-based molecules are involved in AM fungal signaling to actinorhizal hosts. In order to investigate this important question, we have made use of the *in vivo* Ca^2+^ reporter approach to evaluate CSSP activation in epidermal root tissues in response to treatment with crude AM fungal exudates as well as potential Myc CO and Myc LCO signaling molecules. The two evolutionarily distant actinorhizal hosts, *C*. *glauca* and *D*. *trinervis*, were selected as typical examples of either intracellular or intercellular modes of *Frankia* root colonization. Finally, it is important to underline that, in the absence of either morphological root responses or available AM-related reporter genes for the two actinorhizal hosts, this assay is the only technique currently available for assaying CSSP activation in response to potential AM fungal signaling.

Results presented in this article show that, for both actinorhizal hosts, AM fungal exudates are efficient activators of sustained nuclear Ca^2+^ spiking in root atrichoblasts, the cellular targets for AM root entry. Furthermore, sub-micromolar concentrations of CO4 were able to mimic CSSP activation for the two host plants and were significantly more efficient elicitors of Ca^2+^ spiking compared to the tested Myc LCOs. Interestingly, in contrast to the observed CSSP activation in AM-targeted atrichoblasts, CO4 treatment did not elicit Ca^2+^ spiking in root hairs, the epidermal cells of *C*. *glauca* which are not colonized by AM fungi. Finally, although responding robustly to AM fungal signaling with CSSP activation, we show that *D*. *trinervis* atrichoblasts are unresponsive to crude *Frankia* supernatants. Together, these findings underline the cellular specificity of these responses and indicate that actinorhizal host plants represent excellent models for studying endosymbiont-host signaling mechanisms for both intracellular and intercellular colonization.

## Materials and methods

### Plant materials and growth conditions

Transgenic *C*. *glauca* plants expressing the nuclear-localised cameleon reporter gene *p35S*:*NUP-YC2*.*1* [24] were generated *via Agrobacterium tumefaciens* transformation and propagated by taking cuttings as previously described [22]. Transgenic composite plants of *D*. *trinervis* (also now known as *Ochetophila trinervis*) expressing the *p35S*:*NUP-YC2*.1 reporter in roots were obtained using ARquaI *A*. *rhizogenes*-mediated transformation according to the protocol described in [25]. Transgenic plants were grown in liquid Broughton and Dillworth (BD) medium [26].

### Preparation of germinated spore exudates of *Rhizophagus irregularis* and *Frankia* supernatants

*R*. *irregularis* (Agronutrition, Labège, France) germinated spore exudates (GSEs) were kindly provided by Soizic Rochange and prepared as described in [18] by incubating 125,000 sterile spores in 40 ml of H_2_O for 7 d in the dark. These GSEs were then concentrated 40-fold and applied to freshly excised root segments. Cell-free supernatants were prepared from “induced” cultures of *Frankia* strains which nodulate either *C*. *glauca* (*Frankia* CcI3, referred to in this article as *F*. *casuarinae* [27]) or *D*. *trinervis* (*Frankia* BCU110501, referred to in this article as *F*. *discariae* [28]). For both *F*. *casuarinae* (Fci) and *F*. *discariae* (Fdi) supernatants the post-fix “i” refers to the induction of the *Frankia* cultures by addition of the respective host plant exudates 5 days before harvesting as described in [22, 29]. These induced *Frankia* supernatants were applied to root segments at a 100-fold dilution.

### Chitin-based elicitors

Chitotetraose (CO4) was purchased from Megazym (LIBIOS, Pontcharra, France). An aqueous stock solution of 10^−3^ M CO4 was diluted to either 10^−6^ or 10^−8^ M. Sulphated and non-sulphated Myc LCOs were kindly provided by Fabienne Maillet and prepared as described in [18]. Since Myc LCO solutions were prepared in diluted acetonitrile, comparative experiments with chitotetraose were performed using CO4 diluted with identical acetonitrile concentrations (0.5% acetonitrile for 10^−6^ M CO4 and 0.005% acetonitrile for 10^−8^ M CO4).

### Nuclear Ca^2+^ spiking responses in *C*. *glauca* and *D*. *trinervis* roots expressing the *p35S*:*NUP-YC2*.*1* reporter

Assays for nuclear Ca^2+^ spiking were performed using young lateral root segments (0.5 to 0.8 cm long) freshly excised from hydroponically-grown transgenic plants expressing the *p35S*:*NUP-YC2*.*1* cameleon reporter. Root explants were placed in a microchamber and treated with 150 μl of the solution to be tested as described in [18]. Confocal FRET-based ratio imaging for detecting and plotting relative changes of nuclear Ca^2+^ levels corresponding to YFP-to-CFP fluorescence intensity changes over time was performed according to [24] using a Leica TCS SP2 AOBS confocal laser-scanning microscope. Imaging was performed on both atrichoblasts and trichoblasts (root hairs) in the case of *C*. *glauca*. In contrast, Ca^2+^ spiking responses were only studied in *D*. *trinervis* atrichoblasts since few root hairs developed on *D*. *trinervis* roots under our growth conditions. It should be underlined that despite limited root hair formation on hydroponically-grown *D*. *trinervis* roots, efficient intercellular *Frankia* colonization and nodulation can be observed on these roots [e.g. 25]. Experiments were repeated several times for each treatment using independent roots (detailed in **S1 Table**).

## Results

### Initial outer root colonization by AM fungi is intracellular for both *C*. *glauca* and *D*. *trinervis*

**Fig 1:**
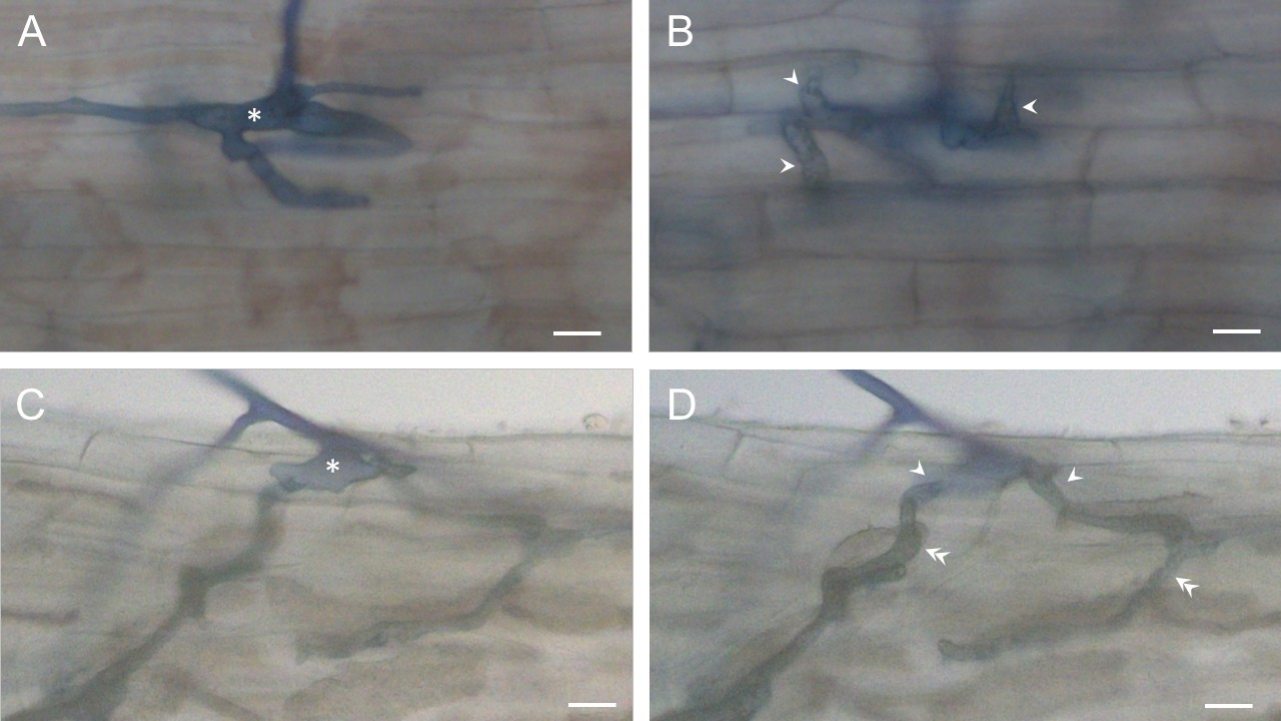
*Rhizophagus irregularis* hyphae enter actinorhizal host roots intracellularly *via* atrichoblasts. Roots of *C*. *glauca* (A, B) and *D*. *trinervis* (C, D) plants were observed 15d after inoculation with spores of *R*. *irregularis*. Asterisks mark AM fungal hyphopodia formed on the root surface at sites of hyphal root entry. Single and double arrowheads indicate transcellular hyphae that cross respectively epidermal and outer cortical cells. The two focal planes were selected to highlight either the surface hyphopodia (A, C) or the transcellular hyphae (B, D). To facilitate appreciation of these data, superimposed images (C and D) are presented as an animated Gif in S1 Fig, as well as an animated Gif of a second site of AM fungal colonization of a *D*. *trinervis* root (S2 Fig). Bars: 10 μm.

Late stages of AM fungal colonization have been described for both *C*. *glauca* [29–32] and *D*. *trinervis* [33]. In both cases, the presence of intracellular symbiotic arbuscules located within inner root cortical cells, accompanied by axially growing intercellular fungal mycelia is consistent with classical Arum-type root colonization [34]. Since the filamentous nitrogen-fixing *Frankia* microsymbiont penetrates the root outer tissues of these two actinorhizal hosts *via* contrasting mechanisms, it was essential to examine the initial stages of AM fungal root entry for both species. The images shown in **Fig 1** and the accompanying Gif animations (**S1 and S2 Figs**) are consistent with AM hyphopodium formation on the root surface, followed by intracellular hyphal root penetration of the outer root tissues for the two host plants. This suggests that the AM fungus crosses the epidermal and outer cortical cell layers *via* a transcellular apoplastic mechanism analogous to what has already been observed for both legume and non-legume hosts [35–37]. Furthermore, initial AM fungal entry for the two actinorhizal hosts occurs exclusively *via* epidermal atrichoblasts, again similar to earlier findings for other AM hosts [38]. Thus, despite striking differences in the modes of *Frankia* root colonization for *C*. *glauca* and *D*. *trinervis*, it is likely that comparable cellular mechanisms operate during initial root entry of the AM fungal partner.

### AM fungal spore exudates trigger sustained Ca^2+^ spiking in the root epidermis of actinorhizal host plants

Our next objective was to investigate the capacity of AM fungal signals to activate the CSSP in actinorhizal host epidermal tissues, focusing primarily on AM-targeted atrichoblast cells. For this we exploited transgenic plant tissues expressing the cameleon sensor Nup-YC2.1 in order to detect nuclear-associated Ca^2+^ oscillations characteristic of CSSP activation [24]. In the case of *C*. *glauca*, stable Nup-YC2.1-expressing lines were already available as described in [22]. For *D*. *trinervis*, composite plants expressing Nup-YC2.1 in root tissues were obtained *via A*. *rhizogenes*-mediated transformation (see Materials & Methods; [25,39]).

**Fig 2:**
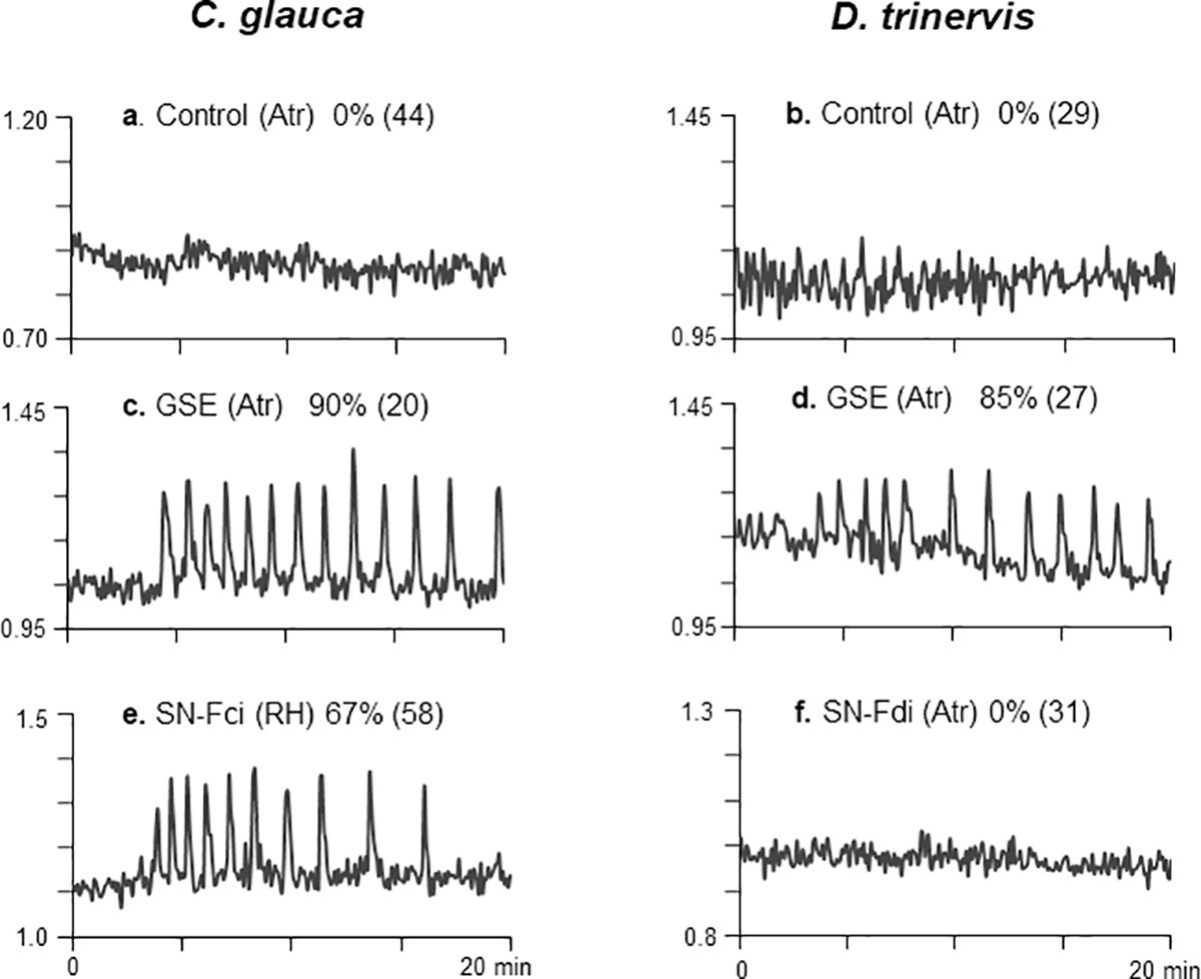
Nuclear Ca^2+^ spiking elicited in root epidermal cells of both *C*. *glauca* and *D*. *trinervis* in response to either AM fungal exudates or *Frankia* supernatants. Freshly excised root segments of *C*. *glauca* and *D*. *trinervis* were treated with either an H_2_O control (a, b), 40x concentrated AM fungal germinated spore exudates (GSE) (c, d) or 100x diluted supernatants of the appropriate induced *Frankia* supernatants (SN-Fci and SN-Fdi: see Materials & Methods) (e, f). Ca^2+^ spiking responses were monitored in either atrichoblasts (Atr) or root hairs (RH) over 20 min periods following root treatment. These experiments show that fungal GSEs elicit sustained spiking responses in both *C*. *glauca* and *D*. *trinervis* atrichoblasts (c, d), the cellular targets for AM colonization. A typical Ca^2+^ spiking response elicited in *C*. *glauca* root hairs by an induced *F*. *casuarinae* supernatant is shown in (e). By comparison, the negative response to induced *F*. *discariae* supernatant treatment of *D*. *trinervis* atrichoblasts is illustrated in (f). Percentages of positively responding cells are indicated for each treatment with the total number of cells examined in brackets.

Initially, we examined whether crude exudates of germinated AM spores were able to activate the characteristic symbiotic nuclear Ca^2+^ spiking response in atrichoblasts of both actinorhizal host plants. Young lateral root segments were harvested from plants growing in liquid medium and treated with a 40x concentrated solution of a *R*. *irregularis* germinated spore exudate (GSE) as described in [18]. Changes in nuclear Ca^2+^ levels within root atrichoblasts were recorded over a 20 min period. Compared with H_2_O controls (**Fig 2A and 2B**), GSE treatment activated sustained nuclear Ca^2+^ oscillations (spiking) in 80–90% of root atrichoblasts, whether from *C*. *glauca* or *D*. *trinervis* roots **(Fig 2C and 2D**). Having established that the CSSP can be activated by AM fungal exudates in the *D*. *trinervis* epidermis, we then evaluated responses to a diluted supernatant from the *F*. *discariae* strain that nodulates *D*. *trinervis* (supernatants from host root exudate-induced cultures were prepared as described in [22]). Strikingly, the *F*. *discariae* (Fdi) supernatant was unable to elicit Ca^2+^ spiking in the *D*. *trinervis* root (**Fig 2F**), despite the fact that intercellular *Frankia* root colonization takes place between atrichoblast cells [10,25]. This contrasts with the situation for *C*. *glauca*, where, as previously shown, supernatants from the induced *F*. *casuarinae* strain (Fci) trigger sustained spiking in *C*. *glauca* root hairs, the target cells for intracellular colonization [22] (**Fig 2E**).

### CO4 mimics the activation of symbiotic Ca^2+^ spiking by fungal GSEs in the two actinorhizal hosts

**Fig 3:**
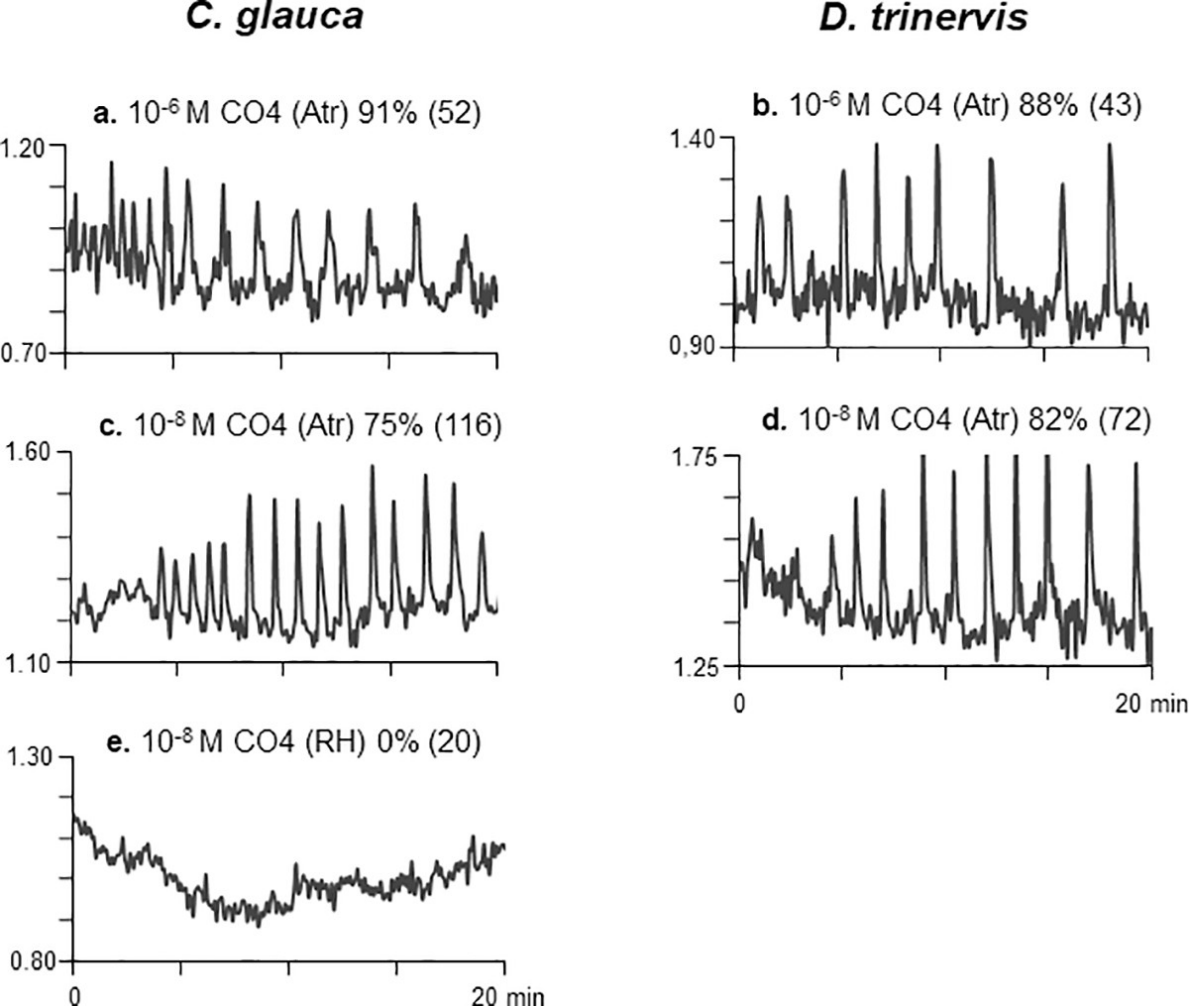
Chitotetraose (CO4) elicits similar nuclear Ca^2+^ spiking to AM fungal GSEs in root atrichoblasts of both actinorhizal hosts. Root segments of both *C*. *glauca* and *D*. *trinervis* were treated with either 10^−6^ M (**a, b**) or 10^−8^ M (**c-e**) CO4, and Ca^2+^ spiking responses monitored in epidermal tissues over 20 min periods. (**a-d**) Both concentrations of chitotetraose elicited sustained spiking responses in atrichoblasts (Atr) of the two hosts resembling those observed with AM fungal exudates (Fig 2). **(e)** In contrast, 10^−8^ M CO4 failed to trigger Ca^2+^ spiking in *C*. *glauca* root hairs (RH). Percentages of positively responding cells are indicated for each treatment with the total number of cells monitored in brackets. Note that these figures combine all CO4 treatments (see S1 Table).

The short-chain chitin oligomer chitotetraose (CO4) is able to activate the AM-dependent CSSP of both legume and non-legume hosts at sub-micromolar concentrations [18,19]. We therefore tested the capacity of CO4 to trigger nuclear Ca^2+^ spiking in root atrichoblasts of *C*. *glauca* and *D*. *trinervis* at both 10^−6^ M and 10^−8^ M. Data presented in **Fig (3A–3D)** show that both concentrations of CO4 elicit pronounced and sustained Ca^2+^ spiking responses similar to those observed with AM fungal GSEs (**Fig 2**). Percentages of responding cells ranged from 75–90% and were marginally higher when CO4 was added at μM levels. In contrast to AM fungi, *F*. *casuarinae* initially colonizes *C*. *glauca via* root hairs, and as shown above, *Frankia* supernatants elicit Ca^2+^ spiking in these epidermal cells (**Fig 2E**). For this reason, we also evaluated the reactivity of *C*. *glauca* root hairs to exogenous 10^−8^ M CO4. Out of the 20 root hairs examined (from three independent root segments), not a single cell responded positively to the Myc CO elicitor (**Fig 3E**). These findings therefore demonstrate that chitotetraose can mimic the activation of the CSSP by AM fungal GSEs in root atrichoblasts of both actinorhizal host plants, but is not able to trigger nuclear Ca^2+^ spiking in *C*. *glauca* root hairs.

### Myc LCOs are less efficient elicitors of nuclear Ca^2+^ spiking in actinorhizal hosts as compared to CO4

**Fig 4:**
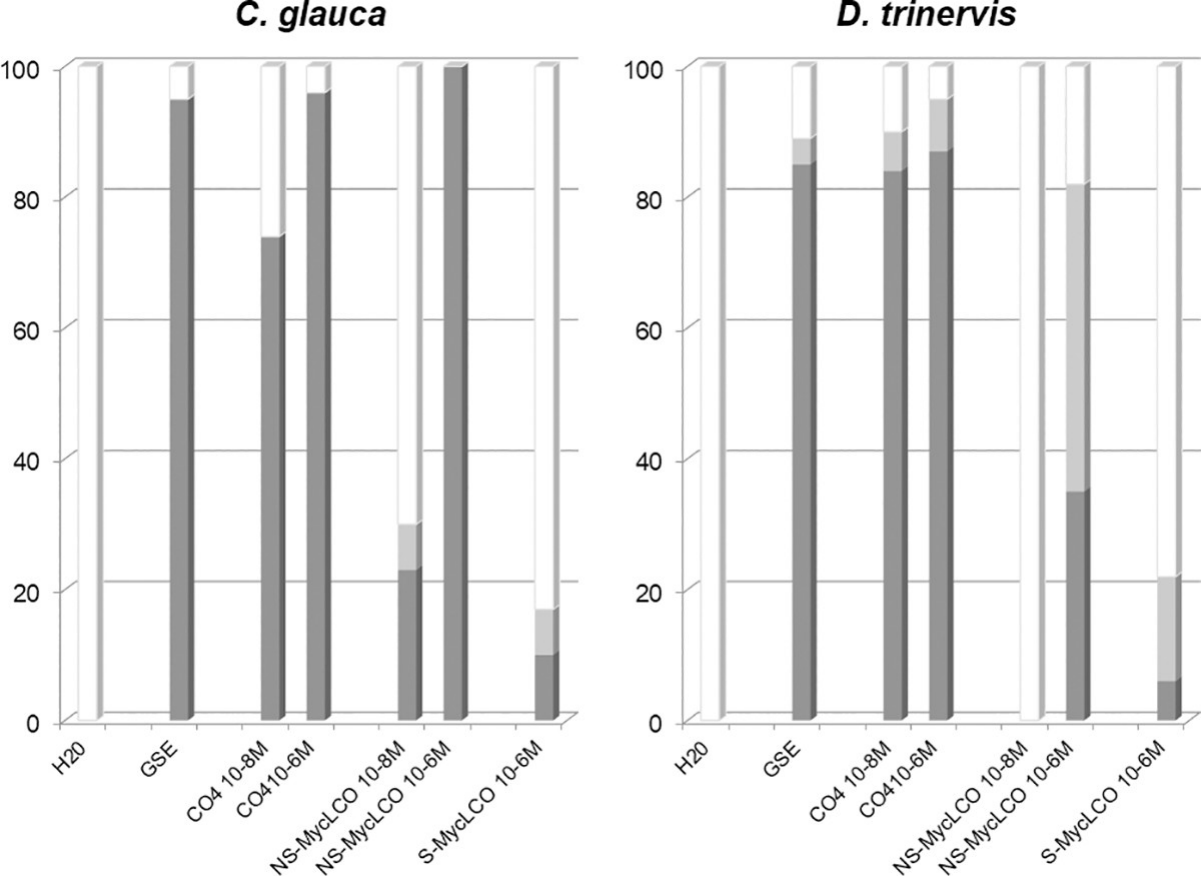
Chitotetraose is a more efficient elicitor of Ca^2+^ spiking in root atrichoblasts of both *C*. *glauca* and *D*. *trinervis* by comparison with Myc LCOs. Root segments of both actinorhizal host plants were treated with either GSEs (40x concentrated), CO4 (10^−8^ & 10^−6^ M), non-sulphated (NS)-Myc LCOs (10^−8^ & 10^−6^ M), or sulphated (S)-Myc LCOs (10^−6^ M). For each treatment the dark grey bars indicate the percentage of atrichoblast cells with more than 2 spikes within the 20 min imaging period, the light grey bars 1–2 spikes, and the white bars represent non-spiking cells. See S1 Table for details of the number of cells examined for each condition and note that the data presented in this figure for all the CO4 and Myc-LCO treatments were obtained in the presence of acetonitrile (0.005% for 10^−8^ M dilutions and 0.5% for 10^−6^ M dilutions).

In addition to Myc COs such as CO4, other chitin-based molecules have been identified as potential AM fungal symbiotic signals. LCOs, which structurally resemble rhizobial Nod factors, are also present in AM fungal exudates, and have been shown to activate the CSSP in legume hosts [17–19]. Myc LCOs are present in both sulphated (S-Myc LCOs) and non-sulphated forms (NS-Myc LCOs) in exudates of *R*. *irregularis* [17]. For comparative purposes, we therefore evaluated nuclear Ca^2+^ spiking initiation in both *C*. *glauca* and *D*. *trinervis* root atrichoblasts in response to either S-Myc LCOs or NS-Myc LCOs. These experiments, summarized in **S1 Table** and in histogram form in **Fig 4**, reveal that both sulphated and non-sulphated Myc LCOs are capable of triggering Ca^2+^ oscillations in both *C*. *glauca* and *D*. *trinervis* atrichoblasts (illustrated for NS-Myc LCOs in **S3 Fig**). However, these data also show that Myc LCOs are significantly less active than CO4 in triggering the CSSP in both actinorhizal hosts, and that S-Myc LCOs are even less active than NS-Myc LCOs. In the case of *C*. *glauca*, the difference between CO4 and NS-Myc LCO activity can be best appreciated at the lower 10^−8^ M concentration. Note also that, since spiking was only observed in 20% of atrichoblasts at 10^−6^ M S-Myc LCO, experiments were not performed at the lower concentration of 10^−8^ M. Although a similar overall trend was found for both actinorhizal hosts, the ability to discriminate between CO4 and NS-Myc LCOs appears greater for *D*. *trinervis* by comparison with *C*. *glauca* (**Fig 4).** This difference can also be appreciated in the representative Ca^2+^ spiking profiles shown in **S3 Fig**, where 10^−6^ M NS-Myc LCO elicits a response with lower spiking periodicity in the *D*. *trinervis* epidermis as compared to *C*. *glauca*. Taken together, we conclude that chitotetraose is a more efficient elicitor of CSSP activation in the actinorhizal host root epidermis compared to the two types of Myc LCOs evaluated in this study.

## Discussion

In this article we have investigated early symbiotic signaling between AM fungi and the two distantly-related actinorhizal host plants, *C*. *glauca* and *D*. *trinervis*, examples of either intracellular or intercellular *Frankia* root colonization respectively. Host perception of AM fungal signals was studied for both plants using a nuclear-localized Ca^2+^ reporter to monitor the activation of the conserved symbiotic signal transduction pathway (CSSP) in epidermal root tissues. This approach has revealed that AM fungal spore exudates (GSEs) elicit sustained nuclear Ca^2+^ spiking in root atrichoblasts of both *C*. *glauca* and *D*. *trinervis* (**Fig 2**) when applied at doses equivalent to those used previously for the legume *M*. *truncatula* [18]. This finding is coherent with the observation that, for all three host plants, AM fungal root entry is intracellular, with atrichoblasts as the principal epidermal target (**Fig 1**).

Earlier studies had shown that short-chain chitin oligomers such as CO4/CO5 are present in AM fungal GSEs and furthermore that 10^−8^ M CO4 is sufficient to mimic GSE-elicited Ca^2+^ spiking in *M*. *truncatula* roots [18]. We show here that the same concentration of CO4 is able to trigger spiking responses in root atrichoblasts of both *C*. *glauca* and *D*. *trinervis* which closely resemble those observed following GSE treatment (**Fig 3**). Our data also reveal that, as for *M*. *truncatula*, CO4 is a significantly more active elicitor of the CSSP in roots of both actinorhizal hosts compared to either sulphated or non-sulphated Myc LCOs (**Fig 4**). These findings now add two actinorhizal hosts to the growing list of AM fungal host plants for which 10^−8^ M CO4 is able to elicit nuclear Ca^2+^ spiking [18,19]. Not only are Myc COs active on all the AM hosts examined to date, but in all cases CSSP activation is observed in non-root hair atrichoblasts, the cellular targets for AM colonization. Furthermore, for certain hosts, such as rice [19] and *C*. *glauca* (**Fig 3**) this response is cell-specific by comparison with root hairs (see below). Additional direct evidence favoring the role of Myc COs as signaling molecules in the initial establishment of the AM association has come from the recent finding that AM-defective *Oscerk1* rice mutants are also defective in responding to CO4 [20], thus suggesting that Myc COs are perceived by a novel rice receptor complex comprising OsCERK1 associated with a second CO-binding LysM-containing membrane protein. Future research now needs to be directed towards identifying the corresponding actinorhizal receptor components capable of recognizing these AM fungal signals and thereby triggering the downstream CSSP. Finally, since LCOs do not appear to be involved in early *Frankia*-host signaling in the case of *C*. *glauca* (see below), the observed low-level responses to Myc LCOs are unlikely to be due to crosstalk with a *Frankia*-related pathway. In consequence, we interpret this Myc LCO activity as resulting from less efficient perception by the AM-associated receptor compared to Myc COs such as CO4.

As stated earlier, initial root colonization of *C*. *glauca* by *F*. *casuarinae* occurs intracellularly *via* root hairs [7]. A variety of host bio-assays, including the expression of transgenic Ca^2+^ reporters, have together revealed that the symbiotic factors present within the *Frankia* exudate are unlikely to be either LCOs or COs [22,23], and hence this raises the question of the nature of the receptors for *Frankia* signals in *C*. *glauca*. As expected, *C*. *glauca* root hairs are the principal cellular targets for *Frankia* symbiotic signals [29]. The fact that short-chain COs present in AM fungal exudates can only activate the CSSP in root atrichoblasts (**Fig 3**) leads us to propose that, in the case of *C*. *glauca*, receptors responding to either *Frankia* or AM fungal signals are distinct and specifically localized to the appropriate epidermal target cell. In contrast, the situation differs for *D*. *trinervis*, since initial *Frankia* root colonization occurs intercellularly between adjacent atrichoblasts [10,25]. We show that, although both AM fungal exudates and CO4 trigger nuclear Ca^2+^ spiking in *D*. *trinervis* atrichoblasts, spiking is not observed in these cells in response to *F*. *discariae* supernatants (**Figs 2 and 3**). One possible interpretation of these findings is that pre-infection symbiotic signaling leading to host CSSP activation is absent during the initial stages of *Frankia* colonization of *D*. *trinervis*. This question clearly merits further investigation since there is evidence from legumes that CSSP-related signaling may not be required during rhizobial intercellular “crack entry” invasion of certain host species [40,41]. In conclusion, the results presented in this article indicate that actinorhizal host plants are particularly valuable model systems for comparative studies of endosymbiont-host signaling mechanisms associated with either intracellular or intercellular root colonization.

## Supporting information

S1 Fig Animated Gif of superimposed images from a site of AM fungal colonization of a *D*. *trinervis* root corresponding to (Fig 1C and 1D). (GIF)

S2 Fig Animated Gif of superimposed images from a second site of AM fungal colonization of a *D*. *trinervis* root. (GIF)

S3 Fig Nuclear Ca^2+^ spiking profiles in root atrichoblasts of the two actinorhizal host plants in response to 10^−6^ M NS-Myc LCOs. These representative profiles reflect the lower reactivity of *D*. *trinervis* atrichoblasts to NS-Myc LCOs as illustrated in histogram form in Fig 4.
(PDF)

S1 Table Summary of the Ca^2+^ spiking responses for each treatment including the number of independent roots and the number of cells observed. For each treatment, cells were assigned to one of the three categories presented in histogram format in Fig 4 (non-responding cells, cells with 1–2 spikes/20 min and cells with more than 2 spikes/20 min). Note that, in the case of CO4 treatment, the numbers in brackets refer to the roots/cells treated with CO4 in the presence of acetonitrile (0.005% for 10^−8^ M CO4 and 0.5% for 10^−6^ M CO4; see Materials & Methods) and correspond to the cells assigned to the three spiking categories. Atr = atrichoblast; RH = root hair.
(DOCX)
