# Supplementary figures and images for: Chitotetraose activates the fungal-dependent endosymbiotic signaling pathway in actinorhizal plant species
Source: PLoS One. 2019 Oct 10;14(10):e0223149. doi: 10.1371/journal.pone.0223149 (PMC6786586; doi:10.1371/journal.pone.0223149)

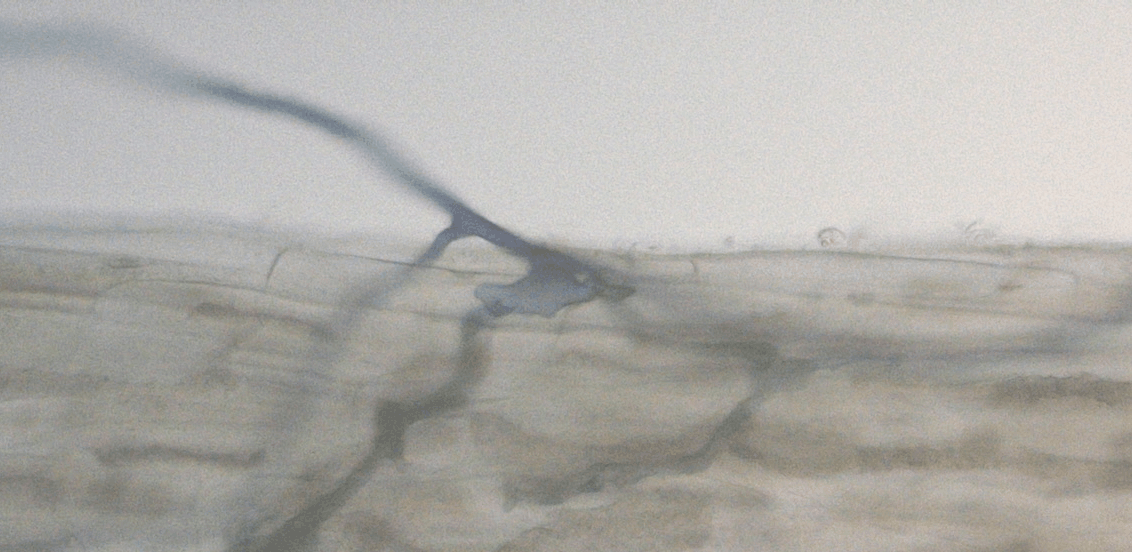

Supplement: S1 Fig — (GIF) [file pone.0223149.s001.gif]

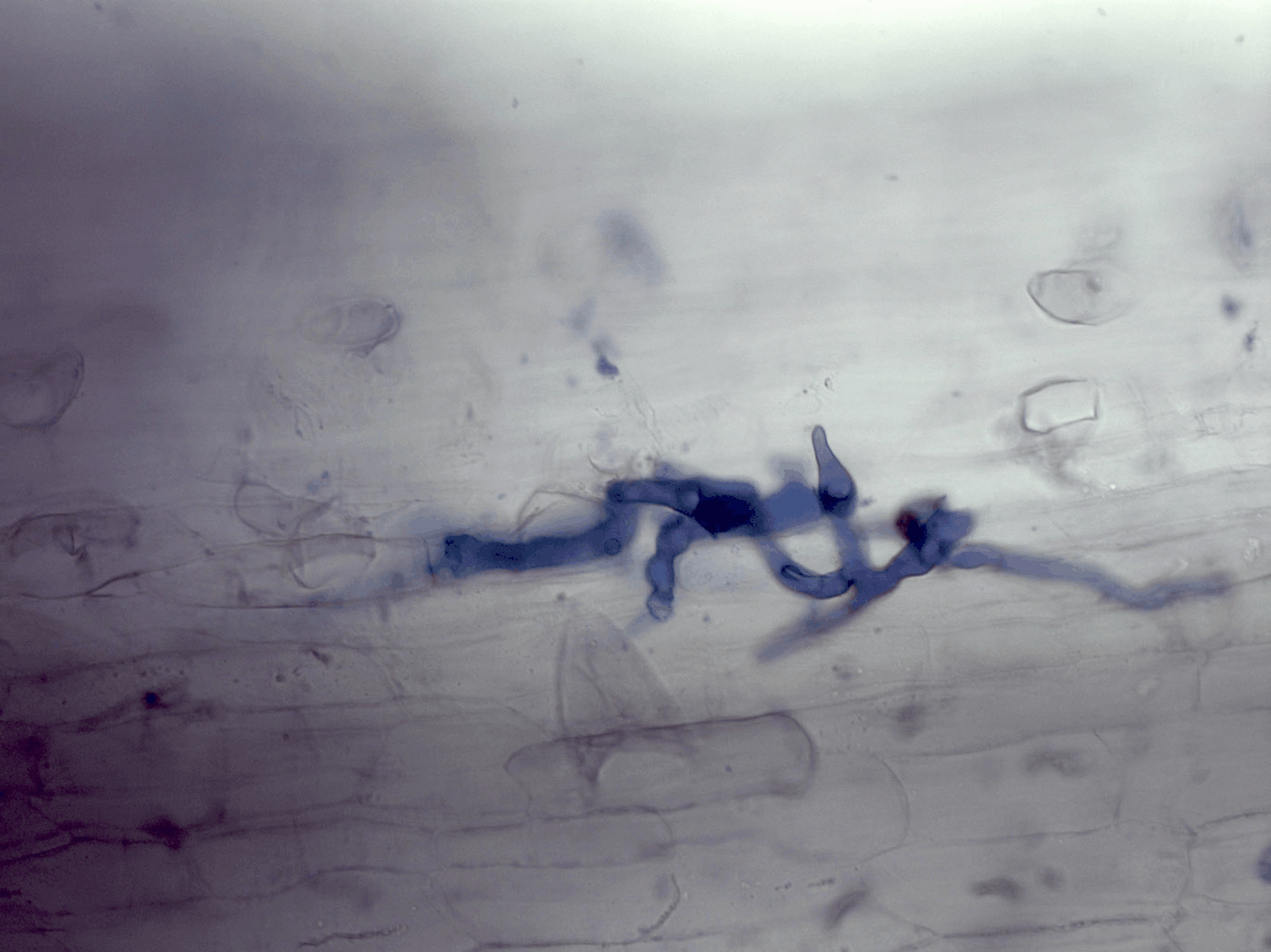

Supplement: S2 Fig — (GIF) [file pone.0223149.s002.gif]
