# Supplementary material for: Chitotetraose activates the fungal-dependent endosymbiotic signaling pathway in actinorhizal plant species
Source: PLoS One. 2019 Oct 10;14(10):e0223149. doi: 10.1371/journal.pone.0223149 (PMC6786586; doi:10.1371/journal.pone.0223149)
Supplement: S3 Fig — These representative profiles reflect the lower reactivity of D. trinervis atrichoblasts to NS-Myc LCOs as illustrated in histogram form in Fig 4. (PDF) [file pone.0223149.s003.pdf]

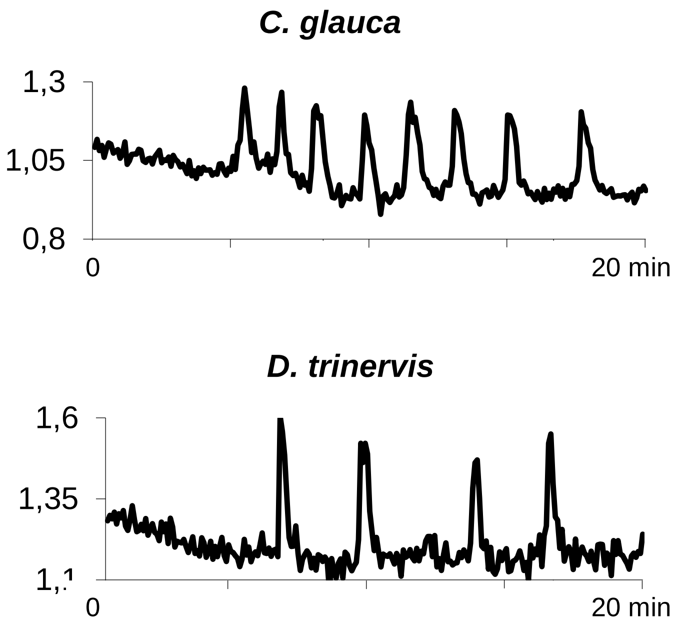

Suppl. Figure 3. Nuclear Ca^{2+} spiking profiles in root atrichoblasts of the two actinorhizal host plants in response to 10^{-6} M NS-Myc LCOs

These representative profiles reflect the lower reactivity of *D. trinervis* atrichoblasts to NS-Myc LCOs as illustrated in histogram form in Figure 4.
