# Supplementary material for: Chitotetraose activates the fungal-dependent endosymbiotic signaling pathway in actinorhizal plant species
Source: PLoS One. 2019 Oct 10;14(10):e0223149. doi: 10.1371/journal.pone.0223149 (PMC6786586; doi:10.1371/journal.pone.0223149)
Supplement: S1 Table — For each treatment, cells were assigned to one of the three categories presented in histogram format in Fig 4 (non-responding cells, cells with 1–2 spikes/20 min and cells with more than 2 spikes/20 min). Note that, in the case of CO4 treatment, the numbers in brackets refer to the roots/cells treated with CO4 in the presence of acetonitrile (0.005% for 10−8 M CO4 and 0.5% for 10−6 M CO4; see Materials & Methods) and correspond to the cells assigned to the three spiking categories. Atr = atrichoblast; RH = root hair. (DOCX) [file pone.0223149.s004.docx]

**S1 Table. Summary of the Ca^2+^ spiking responses for each treatment including the number of independent roots and the number of cells observed.**

*For each treatment, cells were assigned to one of the three categories presented in histogram format in Figure 4 (non-responding cells, cells with 1-2 spikes/20 min and cells with more than 2 spikes/20 min). Note that, in the case of CO4 treatment, the numbers in brackets refer to the roots/cells treated with CO4 in the presence of acetonitrile (0.005% for 10^-8^ M CO4 and 0.5% for 10^-6^ M CO4; see Materials & Methods) and correspond to the cells assigned to the three spiking categories. Atr = atrichoblast; RH = root hair.

| \| ***C. glauca*** \| **Epidermal   cell type** \| **N^o^ of   roots** \| \| \| **N^o^ of cells examined** \| **N^o^ of cells per spiking   category (see Fig. 4)*  0 1-2 ≥3** \| \| --- \| --- \| --- \| --- \| --- \| --- \| --- \| \| **Control** \| Atr \| 4 \| \| \| 44 \| 44 0 0 \| \| **GSE x40** \| Atr \| 2 \| \| \| 20 \| 2 0 18 \| \| **CO4 10^-8^ M** \| Atr \| 12 (**5**) \| \| \| 116 (**47**) \| 12 0 35 \| \| **CO4 10^-6^ M** \| Atr \| 4 (**2**) \| \| \| 52 (**27**) \| 1 0 26 \| \| **NS-MycLCO 10^-8^ M** \| Atr \| 4 \| \| \| 47 \| 33 3 11 \| \| **NS-MycLCO 10^-6^ M** \| Atr \| 4 \| \| \| 40 \| 0 0 40 \| \| **S-MycLCO 10^-6^ M** \| Atr \| 3 \| \| \| 29 \| 24 2 3 \| \| **SN Fci 1/100**  **CO4 10^-8^ M** \| RH  RH \| 12  3 \| \| \| 58  20 \| 19 0 39  20 0 0 \| \| ***D. trinervis*** \|  \| \|  \| \|  \|  \| \| **Control** \| Atr \| 3 \| \| \| 29 \| 29 0 0 \| \| **GSE x40** \| Atr \| 2 \| \| \| 27 \| 3 1 23 \| \| **CO4 10^-8^ M** \| Atr \| 6 (**4**) \| \| \| 72 (**50**) \| 5 3 42 \| \| **CO4 10^-6^ M** \| Atr \| 4 (**3**) \| \| \| 43 (**38**) \| 2 3 33 \| \| **NS-MycLCO 10^-8^ M** \| Atr \| 3 \| \| \| 44 \| 44 0 0 \| \| **NS-MycLCO 10^-6^ M** \| Atr \| 3 \| \| \| 34 \| 6 16 12 \| \| **S-Myc LCO 10^-6^ M**  **SN Fdi 1/100** \| Atr  Atr \| 3  4 \| \| \| 37  31 \| 29 6 2  31 0 0 \| \|  \|  \|  \| \|  \| \|  \| |  |  |  |  |
| --- | --- | --- | --- | --- | --- | --- | --- | --- | --- | --- | --- | --- | --- | --- | --- | --- | --- | --- | --- | --- | --- | --- | --- | --- | --- | --- | --- | --- | --- | --- | --- | --- | --- | --- | --- | --- | --- | --- | --- | --- | --- | --- | --- | --- | --- | --- | --- | --- | --- | --- | --- | --- | --- | --- | --- | --- | --- | --- | --- | --- | --- | --- | --- | --- | --- | --- | --- | --- | --- | --- | --- | --- | --- | --- | --- | --- | --- | --- | --- | --- | --- | --- | --- | --- | --- | --- | --- | --- | --- | --- | --- | --- | --- | --- | --- | --- | --- | --- | --- | --- | --- | --- | --- | --- | --- | --- | --- | --- | --- | --- | --- | --- | --- | --- | --- | --- | --- | --- | --- | --- | --- | --- | --- | --- | --- | --- | --- | --- | --- | --- |
